# Spatial Distribution and Convergence of Agricultural Green Total Factor Productivity in China

**DOI:** 10.3390/ijerph19148786

**Published:** 2022-07-19

**Authors:** Liping Zhu, Rui Shi, Lincheng Mi, Pu Liu, Guofeng Wang

**Affiliations:** Faculty of International Trade, Shanxi University of Finance and Economics, Taiyuan 030006, China; sxlipingzhu@126.com (L.Z.); srui3639@163.com (R.S.); mlc960214@163.com (L.M.); liup_aee@163.com (P.L.)

**Keywords:** agriculture, green total factor productivity, spatial distribution, absolute convergence, conditional convergence, dynamic spatial convergence

## Abstract

The power source, spatial-temporal differentiation and convergence of the growth rate of green total factor productivity in China’s agriculture were analyzed. The Malmquist index was used to measure the growth rate, and the spatial-temporal convergence was tested by σ convergence, absolute β convergence, conditional β convergence and dynamic spatial convergence. The study drew conclusions that the impetus for the intensive growth of green agriculture was insufficient, and the driving force for the growth of agricultural green total factor productivity (AGTFP) in the eastern, western and central region was green technology progress. In addition, AGTFP did not have an absolute σ convergence trend. Dynamic spatial absolute β and conditional β convergence indicated that regional differences were not completely related to regional endowment conditions, and regional green agricultural production was unbalanced. This study provides an important support for regional green development in China’s agriculture.

## 1. Introduction

As a critical supporting division for economic improvement, agriculture has received increased consideration to its development mode. Globally, due to the impact of COVID-19, a growing number of individuals recognize that agricultural products are “good medicine” to remedy infections, thus green and healthy agricultural products and agricultural advancement strategies have been warmly welcomed by everyone [1,2]. According to FAO’s The State of Food Security and Nutrition in the World in 2021, 720–811 million individuals around the world confronted starvation in 2020, an increase of 161 million compared to 2019 [3]. Similarly, nearly 2.37 billion public were incapable of accessing adequate nourishment in 2020, an increase of 320 million in just one year. The main stream of international agricultural development is to convert agricultural structures to furnish nutritious, affordable food for all, and to build more efficient, resilient, inclusive and sustainable agricultural production systems [4]. China, as a populous nation, had 1411.78 million people in the seventh census [5]. The steady increase in population and the transformation of people’s healthy lifestyle promoted the production efficiency and quality improvement of agricultural and rural economy. However, China’s agricultural growth had long been driven by fertilizer, pesticide, agricultural film and diesel oil, and these high-cost resources posed a threat to the quality, health and safety of agricultural products [6]. In addition, agricultural carbon emissions were approaching the peak, thus the green transformation of agriculture became a necessary part of changing the traditional agricultural development mode [7].

Facing the dual pressure of carbon emission constraint and agricultural green economic growth, exploring the relationship between China’s agricultural high-quality development and agricultural green total factor productivity (AGTFP) growth was the key to ensure agricultural green development and even economic development [8,9]. Green, the essence of agriculture and the fundamental way out for agricultural development, was to highlight the promotion of AGTFP in a prominent position. The improvement of TFP played an important role in maintaining sustained and stable economic growth [10]. After China entered the 21st century, there was a new power source for agricultural economic growth. During this period, what were the differences in the overall level and growth trend of AGTFP at different spatial scales? How much were the relevant convergences of the differences? By accurately measuring the AGTFP of different provinces, we could scientifically grasp the differences in agricultural green development in various regions, and effectively solve the resource and environmental problems in the development of agricultural economy. Additionally, the approach would provide theoretical support and a decision-making basis for formulating appropriate regional agricultural green and high-quality coordinated development policies and have important theoretical and practical significance for building the territorial spatial layout of high-quality development.

Under the premise of “promoting agriculture by quality and green”, AGTFP became a popular topic for academics and the government [11]. The AGTFP introduced the environmental pollution and resource consumption factors generated in the process of agricultural production, which could comprehensively measure agricultural economic growth as well as resource and environmental constraints, effectively reflecting the real agricultural production efficiency and agricultural economic development level [12,13]. The AGTFP as an imperative indicator of green agricultural production was mostly characterized by the Malmquist Index [14]. Most studies on regional variations and convergence in AGTFP had been conducted at two levels. One level was the macro level, referring to the country as the research object, and the study covered more than 50 countries or regions around the world. The other level was the meso level, with districts as the research object [15]. There existed σ convergence and β convergence of AGTFP under the constraint of resource environments regarding distinctive areas in China, but the convergence was not stable [16]. Additionally, other studies identified that AGTFP indicated the characteristics of different convergence, and absolute convergence, conditional convergence, club convergence, and panel convergence were also involved in related studies [17]. Overall, it could be discovered that the research on AGTFP had accomplished fruitful research results, providing strong support for practical research, but there were still two deficiencies in its research. First, there were more studies on the evolution of the time dimension of AGTFP, but the studies from the spatial dimension still needed to be reinforced [7,18]. Second, the interpretation of the convergence mechanism of AGTFP was generally examined from a static perspective, and there was a lack of systematic research on the evaluation of its dynamic angle [19,20]. Based on this, the second section of this paper addresses research design and data sources, the next section examines the spatial-temporal and regional differences of AGTFP and its decomposition. The fourth section addresses the convergence analysis of regional differences in AGTFP, and the final section discusses the research conclusions and policy recommendations of this paper.

## 2. Materials and Methods

### 2.1. Agricultural Green Total Factor Productivity

According to the research significance, data availability and related references, the five variables of labor, land, capital, energy and water resources were selected as the input variables of the AGTFP measurement index [21,22,23]. In agricultural production, there was not only the desirable output expressed by the total output value of agriculture, forestry, animal husbandry and fishery, but also accompanied by undesirable output such as non-point source pollution and carbon emissions [24,25]. This logic was in line with the actual agricultural production process. In the procedure of calculating the AGTFP, the constraints of resources and energy should be considered, as well as the harm and impact of environmental pollution. As shown in Table 1, the measurement index system integrating “resources-energy-environment-economy” was constructed.

The agricultural production cycle was long, and the production process was continuous. In the long-term of manufacturing process, the level of technological progress in agriculture was expanding, which contained a driving role in the change of the productivity level [26]. When the Decision-Making Unit (DMU) was panel data, the Malmquist index could replicate the change in productivity better [11]. In the case of undesirable outputs, the Malmquist–Luenberger (ML) index, combining the Malmquist index and the Slacks-Based Measure (SBM), was more adaptable [18,27]. However, the ML index did not have the condition of circularity, and there was the possibility of no solution by linear programming. Numerous improvements had been proposed by scholars to address this drawback. Fare et al. discovered that in the case of constant returns to scale, the input-output could meet the Hicks-neutral technical change, guaranteeing the index circularity to some extent, but this condition was extremely demanding [28]. Pastor and Lovell constructed the production frontier by means of utilizing the examination period of all DMUs as a benchmark and forming the global exponent [29]. The index could effectively avoid the possibility of no solution, meet the requirements of circularity, as well as allow technical regression. The global benchmark enveloped the whole current benchmark into a single set of global production possibilities, serving as a common reference set for each period [30,31]. The sets of production possibilities for current and global benchmarks were shown as follows:

The current benchmark: $P_{C}^{T}\left(x^{t}\right)=\{\left(y^{t},b^{t}\right)|x^{t}\;\mathrm{can}\;\mathrm{produce}\;\left(y^{t},b^{t}\right)\}$
The global benchmark: $P_{G}=P_{C}^{1}\cup P_{C}^{2}\cup \ldots \cup P_{C}^{n}$

The difference between the M-index of the two adjacent periods was that the same production frontier was referenced for each period under the global benchmark, thus a single M-index was calculated. Referring to the research results of Oh, this paper used Global Malmquist–Luenberger (GML) to measure the change of AGTFP in various regions of China, while fully considering the global production possibilities and reflecting the changing trend of different years [32]. Assuming that each province was a DMU, there were 31 DUMs in the *t* inspection period. If provinces used N input elements $x=\left(x_{1},x_{2},\ldots ,x_{N}\right)\in R_{+}^{N}$ in the production process, M desirable outputs $y=\left(y_{1},y_{2},\ldots ,y_{M}\right)\in R_{+}^{M}$ and J undesirable outputs $b=\left(b_{1},b_{2},\ldots ,b_{J}\right)\in R_{+}^{J}$ could be obtained. The expression for the GML index was:

$$GML^{t,t+1}(x^{t},y^{t},b^{t},x^{t+1},y^{t+1},b^{t+1})=(1+D_{G}^{T}(x^{t},y^{t},b^{t}))/(1+D_{G}^{T}(x^{t+1},y^{t+1},b^{t+1}))$$

where $D_{G}^{T}\left(x,y,b\right)=\max\{\beta |y+\beta y,b-\beta b\in P_{G}\left(x\right)\}$ was obtained from the global benchmark production possibility set $P_{G}$. If $GML^{t,t+1}>1$, it indicated that the AGTFP increased; if $GML^{t,t+1}<1$, it meant that the GTFP in agriculture decreased. The GML index could also be decomposed into the Green Technological Efficiency Change (GTEC) and the Green Technological Change (GTC). The TC was used to measure the progress rate at the production forefront, and the TEC was used to measure the degree to which each production unit was approaching the frontier of the existing technology, that is, the efficiency of using technology.

$$\begin{array}{ll} GML^{t,t+1}(x^{t},y^{t},b^{t},x^{t+1},y^{t+1},b^{t+1}) & =\frac{1+D_{G}^{T}(x^{t},y^{t},b)}{1+D_{G}^{T}(x^{t+1},y^{t+1},b^{t+1})}\\ & =\frac{1+D_{G}^{T}(x^{t},y^{t},b)}{1+D_{G}^{T}(x^{t+1},y^{t+1},b^{t+1})}\times [\frac{\left(1+D_{G}^{T}(x^{t},y^{t},b))/(1+D_{G}^{T}(x^{t},y^{t},b)\right)}{\left(1+D_{G}^{T}(x^{t+1},y^{t+1},b^{t+1}))/(1+D_{G}^{T}(x^{t+1},y^{t+1},b^{t+1})\right)}]\\ & =\frac{GTE^{t+1}}{GTE^{t}}\times [\frac{PG_{t+1}^{t,t+1}}{PG_{t}^{t,t+1}}]\\ & =GTEC^{t,t+1}\times GTC^{t,t+1} \end{array}$$

where GTE and GTEC represented the degree and variation of green technology efficiency, respectively. PG was the best gap between the technology frontiers of current and global benchmarks. If GTC>1, green technology progressed; if GTC<1, green technology regressed.

### 2.2. Convergence Test Method Based on Time Trend

(1) Absolute convergence test. Absolute convergence tested whether variations between different economies automatically vanished, including σ convergence and absolute β convergence [19,20].

σ Convergence test. σ convergence tested whether the dispersion degree of GTFP of agriculture in each region was continuously decreasing. If σ dropped gradually, there was a convergence trend between regions. In this study, the standard deviation, coefficient of variation, σ coefficient, Theil index, logarithmic coefficient of variation and Gini coefficient were combined to test the convergence of AGTFP between regions. Since diverse strategies had distinctive sensitivities to the data, the Thiel coefficient was more sensitive to high efficiency level changes, the logarithmic coefficient of variation was sensitive to low efficiency level changes, and the Gini coefficient was more sensitive to moderate efficiency level changes [33,34,35]. Therefore, the mean of the six methods was taken to test the convergence of the cumulative growth rate of AGTFP in China.

Absolute β convergence test. The absolute β convergence test described that the regions with different initial levels of the AGTFP eventually formed a common steady-state level, that was to say, regions with initial levels at a lower rate could achieve similar efficiency levels to those with higher initial levels of the AGTFP by rapid growth [1]. The absolute β convergence test was tuned to the model of Barro and Sala-I-Martin (1995) by the following formula [36].

(1) $$\frac{\ln(y_{it}/y_{i0})}{T}=\alpha +\beta \ln y_{i0}+\mu_{it}$$

where $y_{it}$ and $y_{i0}$ represented the cumulative growth rate of AGTFP in i province of the *t* and initial period. The right side of the expression represented the average annual growth rate of AGTFP in i province during T period, α and β were the estimated coefficient, $\mu_{it}$ was a random error term. If the coefficient value of β was negative and statistically significant, then AGTFP had an absolute β convergence trend.

Furthermore, the rate of convergence could be measured from Mankiw [37].

(2) $$\beta =-(1-e^{-\lambda T})/T$$

In the above equation, it was a convergence analysis of the cross-section, but it was worth noting that the evaluation of the cross-section was hard to reflect the continuity of convergence, and that its result had a strong sensitivity to the time span. Therefore, in order to keep away from singular values that may appear in some time periods and maintain the continuity of time, the absolute β convergence of the AGTFP and its growth rate was performed in the rolling time periods of T = 2, T = 3, T = 5 and T = 12, respectively. The rolling period was finalized, determined as T = 2 in accordance with the size of $R^{2}$. The average of the cumulative growth rate of AGTFP in T = 2 was taken as the value of every period, and then absolute β convergence was examined by distinctive districts.

(2) Conditional β convergence. According to the New Theory of Economic Growth, the economic development status of distinctive districts would be specific in unique periods or in the identical period [38]. If these underlying conditions were controlled, there would be different convergence conclusions. Considering the convergence of the external environment, the conditional β convergence usually tested regions [39]. If conditional β convergence existed, it indicated that the AGTFP steady-state level of each region was related to the resource endowment conditions and that achieving a consistent steady-state level between regions was difficult. There were two methods to test conditional β convergence: one was to fix effects by presetting individuals and time, the other was to appropriately add a control variable to the right side of the absolute β convergence model. The second method held that inter-regional conditional β convergence existed if the regression coefficient was still negative and statistically significant after adding the control variable, so the study used the second approach. To avoid missing important control variables, the level of economic development, agricultural industry structure adjustment, agricultural science and technology input, agricultural infrastructure, rural human capital, industrialization degree, and agricultural energy consumption were selected as control variables in line with the relevant literature [24,40]. Among them, the level of economic development was measured by the per capita gross output [41]. The restructuring of the agricultural industry was calculated by the proportion of the total output of the planting industry in the total agricultural output [42]. Agricultural science and technology input was measured by the stock of agricultural science and technology capital, which needed to be estimated based on the “perpetual inventory method” [43]. The length of highways divided by the administrative area of the province equals agricultural infrastructure [44]. Rural human capital was based on Hall and Jones by using the conversion method of education years, and the degree of industrialization was measured by the total value of non-agricultural output divided by Gross Domestic Product (GDP) [45]. Agricultural energy consumption was characterized by agricultural GDP energy consumption with 100 million yuan. The conditional *β* convergence test model of AGTFP was as follows.
  (3) $$d(\ln y_{it})=\ln y_{it}-\ln y_{i(t-1)}=\alpha +\beta \ln y_{i(t-1)}+\gamma x_{it}+\mu_{it}$$
  where $y_{it}$ is the growth rate of AGTFP in i province of the t period, $x_{it}$ is the control variables, $\mu_{it}$ is the random error term, and α, β, γ are the coefficients to be estimated.

It was remarkable that a sub-regional test was required to judge whether there was convergence within the region to gain a more realistic understanding of regional heterogeneity [16]. Consequently, the 31 provinces were taken as a whole sample in this paper. When examining the regional situation, the regional dummy variable was added at the end of the above formula, and then the fixed effect was used. To avoid being excluded due to the lack of time trend, the interaction terms between the regional dummy variable and AGTFP of the initial period were ultimately added.

### 2.3. Dynamic Spatial Convergence Model

According to spatial econometric theory, the Spatial Durbin Model (SDM), spatial hysteresis mode (SAR) and the spatial error model (SEM) could measure typical forms of spatial associations [46]. Based on traditional absolute β convergence model and conditional β convergence model, the weights matrix (W) characterizing spatial associations was included, obtaining a spatial static β convergence model. However, the model could only investigate the influence of easy-to-measure factors on the dependent variables, and the influence of potentially difficult-to-measure factors such as the cyclical cumulative effect of inter-regional agricultural production factor flows and institutions were easy to be ignored, and these factors had an important impact on AGTFP. Considering this, the interpreted variables of the lag period were included as explanatory variables in the convergence β model. Six models of the AGTFP were constructed on the recommendation of Elhorst, such as dynamic space SDM absolute β convergence model, dynamic space SAR absolute β convergence model, dynamic space SEM absolute β convergence model, dynamic space SDM conditional β convergence model, dynamic space SAR conditional β convergence model and dynamic spatial SEM conditional β convergence model [47]. Among them, the selection criteria for SDM, SAR and SEM models were based on Elhorst [47]. These models used a combination of natural logarithmic function value (Log), maximum likelihood ratio (LR), Schwartz criterion (SC) and Akaike Information Criterion (AIC) to test and judge [48,49,50]. The dynamic SAR model was finally determined as an analytical model, and the fixed effect model was also selected according to the Hausman test [51].

Dynamic space SAR absolute β convergence model was as follows:

(4) $$\ln(y_{it}/y_{it-p})=\alpha +\beta \ln y_{it-p}+\rho \omega \ln(y_{it}/y_{it-p})+\varepsilon_{it}\;\varepsilon_{it}\sim N(0,\sigma^{2})$$

Dynamic spatial SAR conditional β convergence model was as follows:

(5) $$\ln(y_{it}/y_{it-p})=\alpha +\beta \ln y_{it-p}+\rho \omega \ln(y_{it}/y_{it-p})+\gamma x_{it}+\varepsilon_{it}\;\varepsilon_{it}\sim N(0,\sigma^{2})$$

where $y_{it}$ and $y_{it-p}$ indicated the cumulative growth rate of AGTFP in the t and t-p periods, respectively. ω was the spatial weights matrix (using the adjacency matrix), α,β,γ were the regression coefficients. ρ was the spatial correlation coefficient, reflecting the degree and direction of the interaction of GTFP of agriculture between regions, and $\varepsilon_{it}$ was a random perturbation term obeying an independent identically distribution. If β was negative and passes the significance test, the space converges.

### 2.4. Data Sources

Considering the homogeneity and accessibility of the data statistical caliber, the study compiled the relevant data from 31 provinces, municipalities, and autonomous regions in China (excluding Hong Kong, Macao and Taiwan) from 2000 to 2019. The original data involved were taken from official authoritative data. Specifically, they were from the China Statistical Yearbook, China Agriculture Yearbook, China Rural Statistical Yearbook, China Compendium of Statistics 1949–2008, Compendium of agricultural statistics in the 30 years of reform and opening up, China Agricultural Statistical Report, China Population and Employment Statistical Yearbook, China Statistical Yearbook on Science and Technology, China Energy Statistical Yearbook, Educational Statistical Yearbook of China, China Statistical Yearbook on Environment, China Water Resources Bulletin (CWRB) and other statistical yearbooks of some provinces. The original data of agricultural water consumption was taken from the website of the National Bureau of Statistics of China, and the remaining data were compiled by the CWRB of various provinces. Missing data were complemented by the interpolation method.

## 3. Results

### 3.1. Spatial Analysis of Agricultural Green Total Factor Productivity

**Figure 1:**
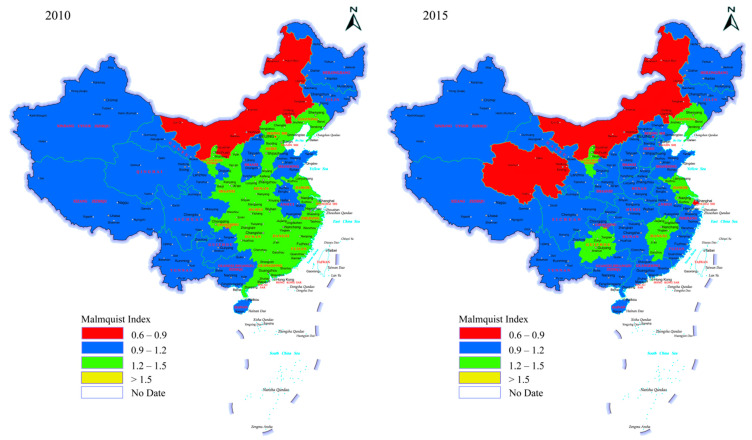

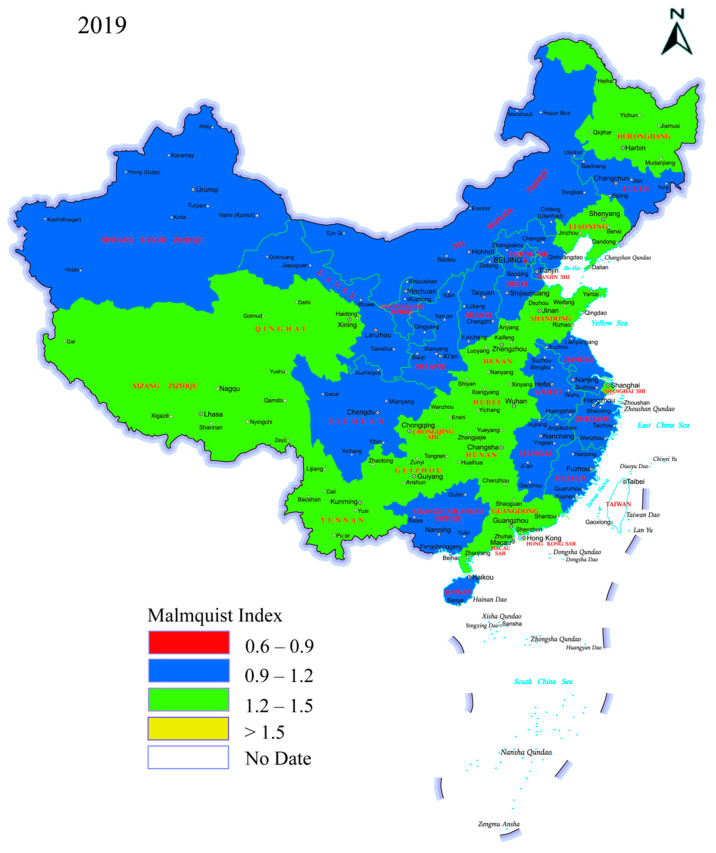
AGTFP in 2010, 2015 and 2019.

From the perspective of the evolution of time, AGTFP showed a significant upward trend between 2010 and 2019 (Figure 1). Taking Inner Mongolia as an example, the range of AGTFP was 0.6–0.9 in 2010–2015, but the range was 0.9–1.2 in 2019, indicating the interval energy level achieved a jump. From 2010 to 2015, agricultural ecological efficiency dramatically dropped, especially in coastal provinces. Numerous provinces in central and eastern areas sharply fell from 1.2–1.5 to 0.9–1.2. However, in terms of the whole nation, the number of provinces in the range of 1.2–1.5 increased significantly concerning AGTFP, showing that the construction of ecological civilization over the years, especially the green development related to agricultural production, achieved remarkable results.

**Figure 2:**
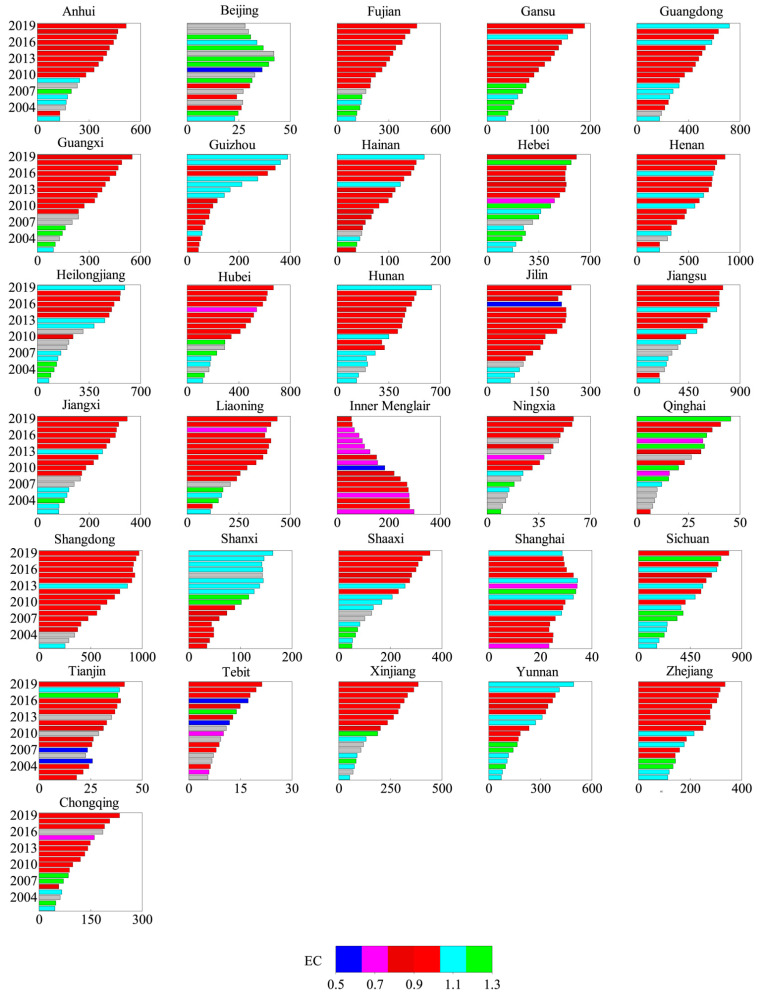
Changes in the composition of TEC values of AGTFP from 2010 to 2019.

**Figure 3:**
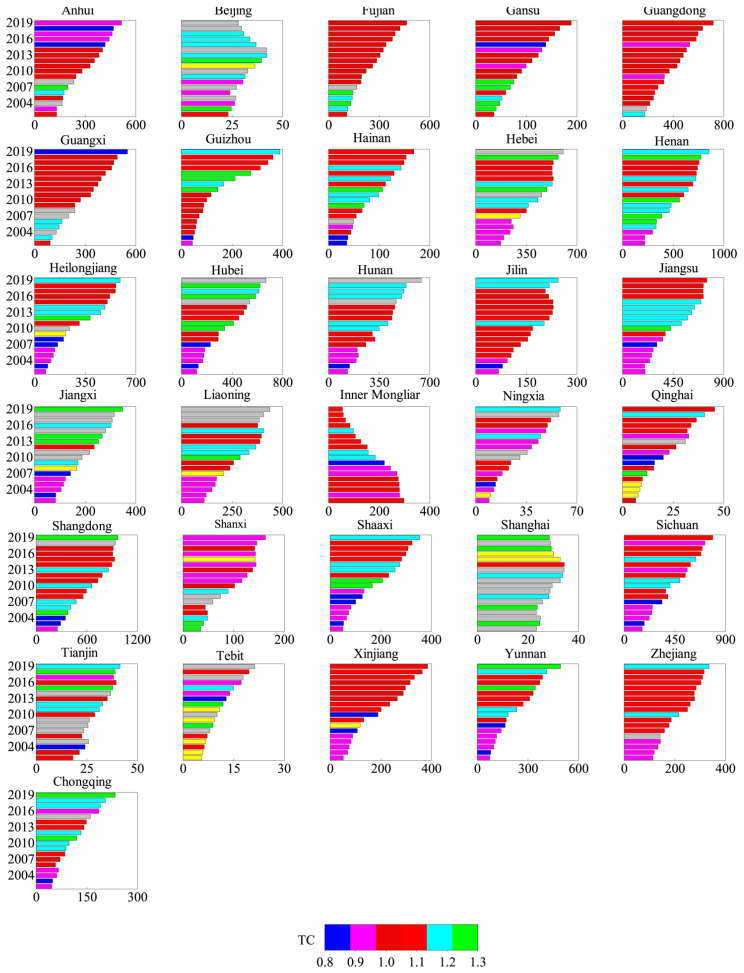
Changes in the composition of TC values of AGTFP in 2010–2019.

By comparing the growth of the eastern, western and central regions (Figure 2), it was obvious that the western region and the central region had achieved continuous growth in AGTFP, with an average annual growth rate of 0.4% and 1.2%, respectively. The growth of the main sales area was driven by the contribution of green technology progress (GTC), which increased by 1.5% and 1.8%, respectively. Specifically, the efficiency of pure green technology (PGT) in the western region had not declined, although there was no improvement in green technology efficiency (GTE). However, the situation in the eastern region was not satisfactory, with an average annual decline of 4.7%, a large decline. In addition, GTC and GTE were declining (Figure 3), reflecting that the pollution and carbon emissions in the eastern region were becoming more serious, and special attention was required to take targeted environmental protection measures. The volatility of AGTFP in the eastern, central and western regions was clear, and the eastern region was the most fluctuate. In particular, the AGTFP in the eastern region showed a rapid growth in the 2015–2019 period.

Overall, 86.7% of provinces with improved AGTFP indicated that China’s agricultural economic growth mode had improved. The AGTFP played a certain role in promoting agricultural economic growth, and thus the factor-driven growth model has changed. The growth rate of the provinces with the largest GTFPC increase reached 5.3%, but the decline rate was 3.8%. It could be predicted that there were some differences in the GTFP growth situation of inter-provincial agriculture. In terms of TC growth, the provinces with TC improvement accounted for 87%, the largest increase and decline in TC were 4.8% and 2.4%, respectively. This situation was close to that of GTFPC, which stated clearly that green technology advancing TC was the key driving source of the growth of AGTFP. Judging from the growth of EC, the improved provinces accounted for only 25%, so there was still much room for improvement in EC in most provinces.

### 3.2. Convergence Analysis of GTFP of Agriculture

#### 3.2.1. Absolute σ Convergence

There was no significant σ convergence between provinces in the cumulative growth rate of AGTFP. The divergence of the growth rate in the eastern, central and western areas showed clear fluctuations, with the eastern region fluctuating the most intensely, followed by the western region, and the central region fluctuating more moderately. The rise and fall of the σ convergence values in the three functional zones, however, reflected no significant σ convergence trend between the provinces. Based on the neoclassical economic growth theory, the less efficient region had a high rate of improvement, so the inter-provincial gap of AGTFP would be gradually narrow, but the test results do not prove this. The reason was that the agricultural technology promotion system was a critical organizational guarantee in the process of transformation and application of scientific and technological achievements, and played a key role in boosting technology diffusion, but the new extension system had not yet been established, and there were problems such as serious administration and weak service, resulting in extension services lagging far behind technology and market demand. Moreover, the level of technology involving environmental pollution and resource-saving green technology in this study, was insufficient and its promotion was extremely weak, leading to difficulty in the distribution of green technology. The higher efficiency zone of AGTFP maintained a higher efficiency level, the lower efficiency area was difficult to quickly imitate learning, and the gap between regions continued to expand for a long time.

#### 3.2.2. Absolute β Convergence

AGTFP showed a significant absolute β convergence trend throughout the study period, and the convergence rate was small. The significant convergence trend in the eastern, central and western districts was clear (Table 2), among which the eastern region had the highest convergence rate, reaching 4.4%. It also passed the significance level test of 1% in different time periods and had an absolute β convergence trend, but the convergence rate varied slightly in each period. It was found that the convergence rate continuously descended from 17.8% to 4.5% in 2002–2005 and 2006–2010. From 2011 to 2015, the rate increased by 11.1%, but this trend was quickly replaced by a decline of 4.4% in 2016–2019. Overall, the gap in the growth rate intra-regional AGTFP was narrowed, and the area with higher growth rates at the beginning of the period slightly declined with time, eventually realizing intra-regional homeostasis.

#### 3.2.3. Conditional β Convergence

The cumulative growth rate of AGTFP in China passed the significance test of 1% during the studied period. In terms of distinctive regions, the condition β convergence test of 1% was demonstrated in the eastern, central and western regions. There was a significant conditional β convergence state in the cumulative growth rate of China’s AGTFP. In addition, there were also significant conditional β convergence states in the eastern, central and western regions. Provinces in different regions tended to their own steady-state levels over time, and the diversity between areas lied in different convergence rates. Therefore, for the sake of the accomplishment of the coordinated progress of the cumulative growth rate of inter-regional and inter-provincial AGTFP, it was needed to exert influence on the steady-state level of each region and province. If the gap of steady-state level lessened, the inter-regional and inter-provincial gap would shrink, which would achieve the coordinated development of regional agriculture.

#### 3.2.4. Dynamic Spatial Convergence

As could be seen from the testing results of the dynamic space SAR absolute β convergence, the whole country and the subregions passed the significance inspection. Compared with the static absolute β convergence test results, the β coefficient shifted from negative to positive, and the varying degree of the coefficients varied. Through regional comparison, the β coefficients of the whole country, the eastern, central and western regions were positive, reflecting the cumulative growth rate of AGTFP presented divergent state for a long time. This trend appeared as a state of “the faster the faster, the slower the slower”, indicating that the gap in the growth rate of AGTFP between regions would widen. The western and central regions took on significant club β convergence, but the eastern regions presented club divergence. The apparent change in the direction of the absolute β coefficient after the inclusion of the first-order spatial lag factors reflected the regional flow of the underlying factors on the cumulative growth rate of AGTFP. If these factors were ignored, the wrong conclusion would be drawn that the growth rate of AGTFP naturally converged. The national or the eastern, central and western spatial correlation coefficients passed the significance test of 1% and were positive, indicating that spatial factors had a positive role in promoting the cumulative growth rate of AGTFP. To be specific, Spatial Neighborhood Relation took effect on the promotion of agricultural green technology and knowledge dissemination, and the relevant areas could coordinate and share agricultural high-quality resource elements to maximize the efficiency of resource allocation and improve the growth rate of AGTFP. Considering distinctive time periods, the absolute β convergence coefficients of the cumulative growth rate of AGTFP measured by the dynamic spatial lag convergence model was positive in 2002–2005, 2006–2010, 2011–2015 and 2015–2019. Compared with the results concluded by the static model, the direction of the absolute β convergence coefficient was reversed, showing that the growth rate of AGTFP did not have an absolute β convergence trend during the investigation period and the sub-time periods. From the angle of the coefficient and significance of the spatial correlation coefficient ρ, there was a dramatic spatial correlation in 2006–2010 and 2016–2019, indicating that spatial connection between Chinese agricultural production regions strongly lacked before. This was because the inter-provincial administrative boundaries and regional lock-in effects formed a barrier to the spatial spillover effect of the growth rate of AGTFP. After 2016, with the advancement of regional economic integration process, the inter-provincial spatial correlation had been increasingly strengthened, and the spatial spillover effect of AGTFP’s growth had been clear.

Through analyzing the cumulative growth rate of AGTFP of the dynamic space SAR condition β convergence, we came to a conclusion that the convergence analysis results had large changes. One change was that the β coefficient shifted from negative to positive, and the other was that the R^2^ fitting coefficient was evidently raised. This change showed that the regional differences in the cumulative growth rate of AGTFP were not only related to the initial resource endowment conditions of the region, but also directly affected by the environmental policy, agricultural green production technology and factor flow and other implicit factors. The spatial correlation coefficients ρ were positive in the western and central regions, of which the 1% significance level test was not dramatic; furthermore, ρ in the national and eastern regions were not dramatic either, stating clearly that the growth rate of AGTFP in the western and central regions had a positive spatial spillover effect. Additionally, the eastern, central and western regions had a significant club divergence trend, so the administrative barriers in various regions needed to be deeply broken, promoting inter-regional agricultural exchanges, cooperation and sharing and attaining green and coordinated development of regional agriculture. Compared with different time periods, the conditional β convergence coefficients of the growth rate of AGTFP were significantly positive in 2002–2005, 2006–2010, 2011–2015 and 2016–2019, and the growth rate did not have a conditional β convergence representation. The spatial correlation coefficients all passed the significance test of 1% and were positive, and the spatial correlation coefficient kept increasing with time, indicating that the spatial spillover effect of the growth rate of AGTFP was increasing. It was critical to further promote the exchange of agricultural green production activities between regions. Regions with a higher growth rate of AGTFP played a demonstrative role in promoting the spread of the “trickle effect” and driving the continuous improvement of the growth rate of AGTFP in China.

## 4. Discussion

Regarding the spatial distribution and convergence of AGTFP in China, the following three feasible suggestions were made. Firstly, paying attention to the spatial pattern of AGTFP and formulating a regionally differentiated growth strategy. Highlighting the functional attributes of the eastern, central and western regions and moderately concentrating on production is crucial. Furthermore, the space spillover effect devotes its impact on improving the demonstration role of radiation. In the long term, the growth rate of the western and central regions was close, but the gap was apparent in contrast to eastern regions. Provinces with lower AGTFP in central and western China should use the opportunities to learn from the frontier experience in the east, for instance, to narrow the gap by updating the management experience and technical exchanges. The aim of attaching importance to the convergence effect is promoting the coordinated growth of AGTFP between regions.

Secondly, administrative boundaries could be weakened, and cross-regional agricultural green cooperation mechanisms could be established. The advice could effectively enhance the complementary advantages of agricultural green growth between regions by building the space correlation network, constructing the overflow channel of green knowledge and technology, optimizing the coordinated allocation of inter-regional resources, such as scientific and technological investment and high-quality rural human capital, highlighting the regional differences and maintaining moderate cross-regional liquidity. In addition, it could perfect several related institutional guarantees. Establishing the interest mechanism of the cross-regional effective cooperation and shared green growth is required to comprehensively use a “combination fist”, including the laws, policies, economies and societies, to balance the interests of various regions. It is critical to provide effective guarantees and constraints for inter-regional agricultural cooperation and green growth at the institutional level.

Thirdly, improving the incentive mechanism and build a platform for cooperation. Specific methods involve the establishment of agricultural green development funds to help agricultural green research and development institutions; the repair of the agricultural intellectual property rights system; the encouragement and protection of the agricultural green innovation achievements; the encouragement of the foreign technology exchanges to promote local green technology innovation; the construction of inter-regional cooperation and dialogue platforms such as technical exchange centers; and the development of the diversified platforms for sharing government, production, learning and research. The above measures could narrow the gap between “agriculture and science and technology” and realize the connection between agricultural green technology and agricultural green growth.

## 5. Conclusions

This paper used the Malmquist index to calculate the growth rate of China’s AGTFP and analyzed the power source of its growth from the national, eastern, central and western and inter-provincial scales. The convergence of regional differences in China’s AGTFP from the two dimensions of time and space was revealed, and the main conclusions were shown as follows:

Under the existing technical level, the resources invested in China’s agricultural production process were far from reaching the level of efficient allocation, and the scale effect of agricultural production had not yet reached expectations, so the scale was not economical. The growth rate of AGTFP in China depended on the “single-wheel drive” of green technology progress.

As to the evolution trend of the timing sequence, firstly, the growth rate of AGTFP was slow, and the motivation to rely on it to achieve green intensive growth in agriculture was insufficient. The growth-driven came from the pull of green frontier technology progress, which belonged to the growth-driven type caused by green technology. Secondly, the efficiency of green technology played a role in blocking the catch-up effect of green technology progress, and the green scale efficiency also acted as a reverse role in the efficiency of green technology. China’s agricultural economy was still experiencing extensive growth with a considerable factor being that the technological innovation and technology introduction on AGTFP was offset by extensive input and inefficient utilization of resource factors.

Judging from the spatial differentiation of AGTFP, the growth rate varied significantly in the eastern, central and western region. While the fluctuation in the east was the most intense, with large “Zigzag” volatility; the patterns in western and central areas were basically the same. The comparison of inter-provincial differences indubitably indicated that the proportion of provinces with the improvement of AGTFP accounted for 86.7%. AGTFP made a difference in promoting agricultural economic growth, meaning that the factor-driven growth model had changed and improved China’s agricultural economic growth. There was a certain degree of difference in the growth rate of AGTFP between provinces. The green scale efficiency of different provinces had not improved significantly, which was mostly caused by the difficult improvement of inefficient resource allocation. The key to restricting the growth of AGTFP lied in the decline in the efficiency of green technology, and the fundamental reason for the decline was the blockage of green scale efficiency and green pure technology efficiency, especially the inefficient state of green scale efficiency which became a key problem in the green production process of agriculture.

From the two dimensions of time and space, the regional differences and convergence trends of AGTFP in China were tested and verified. The results of the absolute σ convergence analysis displayed that the cumulative growth rate of AGTFP had no absolute σ convergence trend at the national or block scale, and that the gap between regions was not narrow. The test results of the dynamic spatial absolute β convergence demonstrated changes in symbols and directions compared with that of the static absolute β convergence, indicating that it was difficult to truly characterize the convergence state of the inter-regional differences on AGTFP without considering the cyclical cumulative effect of potential factors, such as the flow of agricultural production factors. Spatial factors emerged as focal points at promoting the cumulative growth rate of AGTFP. For instance, spatial neighborhood relation could push the dissemination of agricultural green technologies and knowledge, so the adjacent zones could share high-quality agricultural resource elements. Moreover, the test results of the dynamic spatial conditional β convergence also revealed signs and orientations compared with that of the static conditional *β* convergence, illustrating that the regional divergence characteristics were clear considering the endowment conditions such as the economic development level of each region. The above demonstration could explain in detail that the regional disparity was not entirely related to endowment conditions, and the role of potential factors such as the flow of agricultural production factors on distinctive districts could not be ignored. The cyclical cumulative effect of factors promoted the spatial agglomeration state of AGTFP through the self-realization mechanism, and the regional agricultural green production was unbalanced. The spatial spillover effect of the growth of AGTFP was increasing with time, which needed to further promote the “trickle-down effect”.

## Figures and Tables

**Table 1:** Measurement index system concerning AGTFP.

Metric Category	Variable Name	Metric Name	Evaluation Indicators	Unit
Input indicators	Labor input	Labor	Number of employees in agriculture, forestry, animal husbandry and fishery	10,000 people
	Land input	Land	Area sown to crops and area of aquaculture	thousand hectares
	Capital investment input	Machine	Total power of agricultural machinery	10,000 kilowatts
		Fertilizer	The amount of agricultural chemical fertilizer and the amount of organic fertilizer applied	10 kilo-tons
		Pesticide	Amount of pesticide use	10 kilo-tons
		Agricultural film	Agricultural film use	Ton
	Energy input	Diesel oil	Agricultural diesel usage	10 kilo-tons
		Electricity	Electricity consumption in agriculture	kWh
	Water resources input	Water	Agricultural water consumption	100 million cubic meters
Output indicators	Desirable output	AgiGDP	The total output value of agriculture, forestry, animal husbandry and fishery	100 million yuan
	Undesirable outputs	AgiNPSP	Emissions of agricultural non-point source pollution	10,000 cubic meters
		AgiE	Agricultural carbon emissions	10 kilo-tons

**Table 2:** Table of absolute β convergence coefficients.

Coefficient	Sub-Regions				Divided into Time Periods			
	Nationwide	Eastern	Western	Central	2002–2005	2006–2010	2011–2015	2016–2019
β	−0.025 *** (0.005)	−0.024 *** (0.007)	−0.027 *** (0.005)	−0.026 *** (0.009)	−0.118 *** (0.054)	−0.040 *** (0.017)	−0.085 *** (0.031)	−0.037 *** (0.038)
a	−0.003 (0.001)	−0.003 (0.002)	−0.002 ** (0.001)	−0.001 (0.002)	−0.008 (0.011)	−0.001 ** (0.002)	−0.002 (0.001)	0.011 *** (0.002)
R^2^	0.964	0.94	0.963	0.992	0.79	0.806	0.915	0.802
λ	0.038	0.036	0.045	0.042	0.178	0.045	0.111	0.044

Note: **, *** represent significant of 5% and 1%.

## Data Availability

Some or all data and models that support the findings of this study were available from the corresponding author upon reasonable request.

## References

[1] Chen Y., Fu W., Wang J. (2022). Evaluation and Influencing Factors of China’s Agricultural Productivity from the Perspective of Environmental Constraints. Sustainability.

[2] Yu D., Liu L., Gao S., Yuan S., Haipeng C., Qianling S. (2022). Impact of Carbon Trading on Agricultural Green Total Factor Productivity in China. J. Clean. Prod..

[3] (2021). The State of Food Security and Nutrition in the World. https://www.fao.org/state-of-food-security-nutrition/en.

[4] Liu Y., Sun D., Wang H., Wang X., Yu G., Zhao X. (2020). An Evaluation of China’s Agricultural Green Production: 1978–2017. J. Clean Prod..

[5] Akimov A., Gemueva K.A., Semenova N.K. (2021). The Seventh Population Census in the PRC: Results and Prospects of the Country’s Demographic Development. Her. Russ. Acad. Sci..

[6] Long D.J., Tang L. (2021). The Impact of Socio-Economic Institutional Change on Agricultural Carbon Dioxide Emission Reduction in China. PLoS ONE.

[7] Xu X., Huang X., Huang J., Gao X., Chen L. (2019). Spatial-Temporal Characteristics of Agriculture Green Total Factor Productivity in China, 1998-2016: Based on More Sophisticated Calculations of Carbon Emissions. Int. J. Environ. Res. Public Health.

[8] Liu D., Zhu X., Wang Y. (2021). China’s Agricultural Green Total Factor Productivity Based on Carbon Emission: An Analysis of Evolution Trend and Influencing Factors. J. Clean Prod..

[9] Wang Y., Xie L., Zhang Y., Wang C., Yu K. (2019). Does FDI Promote or Inhibit the High-Quality Development of Agriculture in China? An Agricultural GTFP Perspective. Sustainability.

[10] Chen Y., Miao J., Zhu Z. (2021). Measuring Green Total Factor Productivity of China’s Agricultural Sector: A Three-Stage SBM-DEA Model with Non-Point Source Pollution and CO_2_ Emissions. J. Clean. Prod..

[11] He W., Li E., Cui Z. (2021). Evaluation and Influence Factor of Green Efficiency of China’s Agricultural Innovation from the Perspective of Technical Transformation. Chin. Geogr. Sci..

[12] Zhang J., Qu X., Sangaiah A.K. (2018). A Study of Green Development Mode and Total Factor Productivity of the Food Industry Based on the Industrial Internet of Things. IEEE Commun. Mag..

[13] Zhong S., Li J., Chen X., Wen H. (2021). Research on the Green Total Factor Productivity of Laying Hens in China. J. Clean. Prod..

[14] Staniszewski J., Kryszak L. (2022). Do Structures Matter in the Process of Sustainable Intensification? A Case Study of Agriculture in the European Union Countries. Agriculture.

[15] Rahman S., Salim R. (2013). Six Decades of Total Factor Productivity Change and Sources of Growth in Bangladesh Agriculture (1948–2008). J. Agric. Econ..

[16] Huang C., Yin K., Guo H., Yang B. (2022). Regional Differences and Convergence of Inter-Provincial Green Total Factor Productivity in China under Technological Heterogeneity. Int. J. Environ. Res. Public Health.

[17] Lan F., Hu R., Mao H., Chen S. (2021). How Crop Insurance Influences Agricultural Green Total Factor Productivity: Evidence from Chinese Farmers. J. Clean. Prod..

[18] Chen H., Zhu S., Sun J., Zhong K., Shen M., Wang X. (2022). A Study of the Spatial Structure and Regional Interaction of Agricultural Green Total Factor Productivity in China Based on SNA and VAR Methods. Sustainability.

[19] Huang X., Wang X., Chen B., Li F., Su S., Zhang T. (2022). Temporal Trend and Regional Disparity of Agricultural Green Total Factor Productivity in China: Data Envelopment Analysis with Biennial Environmental Technology. Discret. Dyn. Nat. Soc..

[20] Yu C., Wenxin L., Khan S.U., Yu C., Jun Z., Yue D., Zhao M. (2020). Regional Differential Decomposition and Convergence of Rural Green Development Efficiency: Evidence from China. Environ. Sci. Pollut. Res..

[21] Coomes O.T., Barham B.L., MacDonald G.K., Ramankutty N., Chavas J.-P. (2019). Leveraging Total Factor Productivity Growth for Sustainable and Resilient Farming. Nat. Sustain..

[22] Yang H., Liu J.G., Folberth C., Chan F., Marinova D., Anderssen R.S. (2011). Global Agricultural Green and Blue Water Consumptive Uses in the Context of Water Scarcity and Climate Change. Proceedings of the 19th International Congress on Modelling and Simulation (Modsim2011).

[23] Naylor R.L. (1996). Energy and Resource Constraints on Intensive Agricultural Production. Annu. Rev. Energy Environ..

[24] Feng J., Zhao L., Zhang Y., Sun L., Yu X., Yu Y. (2020). Can Climate Change Influence Agricultural GTFP in Arid and Semi-Arid Regions of Northwest China?. J. Arid Land.

[25] Hamid S., Wang K. (2022). Environmental Total Factor Productivity of Agriculture in South Asia: A Generalized Decomposition of Luenberger-Hicks-Moorsteen Productivity Indicator. J. Clean. Prod..

[26] Hu J., Wang Z., Huang Q. (2021). Factor Allocation Structure and Green-Biased Technological Progress in Chinese Agriculture. Ekon. Istraz..

[27] Chi Y., Xu Y., Wang X., Jin F., Li J. (2021). A Win-Win Scenario for Agricultural Green Development and Farmers’ Agricultural Income: An Empirical Analysis Based on the EKC Hypothesis. Sustainability.

[28] Fare R., Grosskopf S., Tyteca D. (1996). An Activity Analysis Model of the Environmental Performance of Firms—Application to Fossil-Fuel-Fired Electric Utilities. Ecol. Econ..

[29] Pastor J.T., Lovell C.A.K. (2005). A Global Malmquist Productivity Index. Econ. Lett..

[30] Gao Q., Cheng C., Sun G., Li J. (2022). The Impact of Digital Inclusive Finance on Agricultural Green Total Factor Productivity: Evidence From China. Front. Ecol. Evol..

[31] Li H., Zhou X., Tang M., Guo L. (2022). Impact of Population Aging and Renewable Energy Consumption on Agricultural Green Total Factor Productivity in Rural China: Evidence from Panel VAR Approach. Agriculture.

[32] Oh D. (2010). A Global Malmquist-Luenberger Productivity Index. J. Prod. Anal..

[33] Serebrenik A., van den Brand M. (2010). Theil Index for Aggregation of Software Metrics Values. Proceedings of the 2010 Ieee International Conference on Software Maintenance.

[34] Castagliola P., Achouri A., Taleb H., Celano G., Psarakis S. (2015). Monitoring the Coefficient of Variation Using a Variable Sample Size Control Chart. Int. J. Adv. Manuf. Technol..

[35] Everett T.J., Everett B.M. (2015). Justice and Gini Coefficients. Polit. Philos. Econ..

[36] Park M.-S. (2010). Capital and Interest in Horizontal Innovation Models. Cambr. J. Econ..

[37] Mankiw N. (1992). The Reincarnation of Keynesian Economics. Eur. Econ. Rev..

[38] Onyimadu C. (2015). An Overview of Endogenous Growth Models: Theory and Critique. SSRN Electron. J..

[39] Cho D., Graham S. (1996). The Other Side of Conditional Convergence|Semantic Scholar. Econ. Lett..

[40] Bachewe F.N., Berhane G., Minten B., Taffesse A.S. (2018). Agricultural Transformation in Africa? Assessing the Evidence in Ethiopia. World Dev..

[41] Zhang Y., Wei J., Wang Y., Tsai S.-B. (2022). An Empirical Study on the Growth of Agricultural Green Total Factor Productivity in the Huanghuai River Economic Zone by Big Data Computing. Math. Probl. Eng..

[42] Wang X., Zhang Y. (2019). Emergy-Based Evaluation of Changes in Agrochemical Residues on the Qinghai-Tibet Plateau, China. Sustainability.

[43] Li H., Tang M., Cao A., Guo L. (2022). Assessing the Relationship between Air Pollution, Agricultural Insurance, and Agricultural Green Total Factor Productivity: Evidence from China. Environ. Sci. Pollut. Res..

[44] Huang H., Zhuo L., Wang R., Shang K., Li M., Yang X., Wu P. (2021). Agricultural Infrastructure: The Forgotten Key Driving Force of Crop-Related Water Footprints and Virtual Water Flows in China. J. Clean. Prod..

[45] Hall R.E., Jones C.I. (1999). Why Do Some Countries Produce So Much More Output Per Worker than Others?. Q. J. Econ..

[46] Anselin L. (1988). Lagrange Multiplier Test Diagnostics for Spatial Dependence and Spatial Heterogeneity-Anselin-1988-Geographical Analysis-Wiley Online Library. Geogr. Anal..

[47] Elhorst J.P. (2012). Dynamic Spatial Panels: Models, Methods, and Inferences. J. Geogr. Syst..

[48] Shi P.D., Tsai C.L. (1998). A Note on the Unification of the Akaike Information Criterion. J. R. Stat. Soc. Ser. B-Stat. Methodol..

[49] Nicolae F., Verjovsky A. (2010). Discrete Schwartz Distributions and the Riemann Zeta-Function. Bull. Braz. Math. Soc..

[50] Nwaogu C., Pechanec V., Vozenilek V. (2019). Responses of Soil and Plants to Spatio-Temporal Changes in Landscape under Different Land Use in Imo Watershed, Southern Nigeria. Arch. Agron. Soil Sci..

[51] Hahn J., Ham J.C., Moon H.R. (2011). The Hausman Test and Weak Instruments. J. Econom..

